# Multisensory and Modality-Specific Influences on Adaptation to Optical Prisms

**DOI:** 10.3389/fnhum.2017.00568

**Published:** 2017-11-22

**Authors:** Elena Calzolari, Federica Albini, Nadia Bolognini, Giuseppe Vallar

**Affiliations:** ^1^Department of Psychology and NeuroMI, University of Milano-Bicocca, Milan, Italy; ^2^Neuro-Otology Unit, Division of Brain Sciences, Imperial College London, London, United Kingdom; ^3^Neuropsychological Laboratory, Istituto Auxologico Italiano, Istituto di Ricovero e Cura a Carattere Scientifico, Milan, Italy

**Keywords:** prism adaptation, multisensory integration, aftereffects, auditory perception, visuo-motor adaptation

## Abstract

Visuo-motor adaptation to optical prisms displacing the visual scene (prism adaptation, PA) is a method used for investigating visuo-motor plasticity in healthy individuals and, in clinical settings, for the rehabilitation of unilateral spatial neglect. In the standard paradigm, the adaptation phase involves repeated pointings to visual targets, while wearing optical prisms displacing the visual scene laterally. Here we explored differences in PA, and its aftereffects (AEs), as related to the sensory modality of the target. Visual, auditory, and multisensory – audio-visual – targets in the adaptation phase were used, while participants wore prisms displacing the visual field rightward by 10°. Proprioceptive, visual, visual-proprioceptive, auditory-proprioceptive straight-ahead shifts were measured. Pointing to auditory and to audio-visual targets in the adaptation phase produces proprioceptive, visual-proprioceptive, and auditory-proprioceptive AEs, as the typical visual targets did. This finding reveals that cross-modal plasticity effects involve both the auditory and the visual modality, and their interactions (Experiment 1). Even a shortened PA phase, requiring only 24 pointings to visual and audio-visual targets (Experiment 2), is sufficient to bring about AEs, as compared to the standard 92-pointings procedure. Finally, pointings to auditory targets cause AEs, although PA with a reduced number of pointings (24) to auditory targets brings about smaller AEs, as compared to the 92-pointings procedure (Experiment 3). Together, results from the three experiments extend to the auditory modality the sensorimotor plasticity underlying the typical AEs produced by PA to visual targets. Importantly, PA to auditory targets appears characterized by less accurate pointings and error correction, suggesting that the auditory component of the PA process may be less central to the building up of the AEs, than the sensorimotor pointing activity *per se*. These findings highlight both the effectiveness of a reduced number of pointings for bringing about AEs, and the possibility of inducing PA with auditory targets, which may be used as a compensatory route in patients with visual deficits.

## Introduction

Prism adaptation (PA) is a technique that, through the use of goggles fitted with prismatic lenses inducing a lateral displacement of the visual field, allows to investigate short-term sensorimotor neuroplasticity in healthy participants (Redding et al., 2005). In a standard PA paradigm, participants are required to make visuo-motor ballistic pointing movements toward a visual target, while looking through prismatic lenses. Typically, the participants’ first pointing movements are deviated toward the direction of the prism-induced visual field displacement (‘direct effect’); however, after repeated manual pointings, the error progressively decreases, and soon participants become able to point correctly to the target (the so-called ‘adaptation,’ which consists in the correction and reduction of the pointing error). Once the prisms are removed, the participants’ pointing movements are still deviated, but in the opposite direction of the previous visual displacement (‘after-effects’). These aftereffects (AEs) are considered the main index of PA, and are usually observed in the somatosensory and visual domains: proprioceptive, visual-proprioceptive, and visual AEs, with the latter being characterized by a shift of the perceptual midline in the direction of the prism-induced deviation of the visual scene (Redding et al., 2005).

Thanks to its directional effects, PA has also been successfully used in the treatment of unilateral spatial neglect (USN). USN is a multicomponent disorder, commonly associated to right brain-damage, defined as the inability to report, respond to, and orient toward stimuli presented in the side of space contralateral to the side of the hemispheric lesion; USN is not due to primary motor and sensory deficits, being brought about instead by a higher-order disorder of spatial attention and representation (Vallar and Bolognini, 2014; Vallar and Calzolari, 2018). A number of clinical manifestations of the USN syndrome can be temporarily reduced after a session of PA (Rossetti et al., 1998; Rode et al., 2001; Pisella et al., 2002; Angeli et al., 2004; Vallar et al., 2006), while repeated sessions of PA may induce long-lasting improvements of USN (Frassinetti et al., 2002a; Serino et al., 2007; Fortis et al., 2010; Vangkilde and Habekost, 2010; Mizuno et al., 2011). Given the rehabilitative potential of PA (for reviews on USN and PA see Barrett et al., 2012; Newport and Schenk, 2012; Jacquin-Courtois et al., 2013), the need of a systematic investigation of this technique has been pointed out, in order to determine the optimal parameters for its clinical application, such as, for instance, the number of adaptation sessions, the type of visuo-motor activity, the magnitude of the prismatic displacement, the exposure duration, or the number of targets during exposure (for a comprehensive review of these issues see Jacquin-Courtois et al., 2013).

In healthy participants, several parameters have been shown to modulate adaptation, such as movement speed (Kitazawa et al., 1997), the presence or absence of visual feedback (Freedman, 1968), the realistic feature of the conflict, with maximal AEs when participants see their actual hand and targets, smaller AEs with a real-time video broadcast, and smallest with an abstract computed generated feed-back (Norris et al., 2001). The simultaneity or close temporal proximity of the execution of the movement and the visual re-afference are most relevant for PA to occur (Hay and Goldsmith, 1973; Kitazawa et al., 1995; see Redding et al., 2005 for a review). In one study, Bornschlegl et al. (2012) showed that pacing with a rhythmic auditory signal, delivered during the pointing movements of PA toward a visual target, may enhance the activation of the PA neural network, likely permitting a multisensory-based selection of more reliable proprioceptive signals for movement control. In this study, the targets were purely visual stimuli, as usually done in PA studies (Redding et al., 2005; Jacquin-Courtois et al., 2013), and the auditory signal (placed to the right and behind the participant) served only as an auditory pacing signal. So far, there is no evidence that PA can be effective when pointing movements are directed to a purely auditory target.

Moreover, auditory stimuli, combined with visual ones, could be also useful to enhance PA. Indeed, multisensory integration is a powerful mechanism for maximizing sensitivity to sensory events. Multisensory integration of auditory and visual stimuli is a common phenomenon in space perception. The principles underlying multisensory integration (the so-called spatial, temporal, and inverse effectiveness rules) have been firstly outlined by neurophysiological and behavioral studies in animals (Stein and Meredith, 1993). Subsequent evidence indicates that similar principles govern multisensory enhancement effects also in humans, improving visual detection (Frassinetti et al., 2002b; Bolognini et al., 2005a; 2013; Convento et al., 2013), visual (Hairston et al., 2003) and auditory localization (Bolognini et al., 2007), and reducing saccadic reaction times (Harrington and Peck, 1998; Hughes et al., 1998; Colonius and Arndt, 2001; Corneil et al., 2002; Arndt and Colonius, 2003). Moreover, it has been found that a sound, spatially and temporally coincident to a visual stimulus, can improve the report of visual events, contralateral to the side of the lesion, in stroke right-brain-damaged patients with left USN (Frassinetti et al., 2002c; Van Vleet and Robertson, 2006), in patients with homonymous hemianopia (Bolognini et al., 2005b; Frassinetti et al., 2005; Passamonti et al., 2009), and in one right-brain-damaged patient with a bilateral impairment of auditory localization with neither USN nor hemianopia (Bolognini et al., 2005c).

The neural correlates of the PA to visual targets, and of the subsequent AEs processes, involve a number of brain structures. An early neuroimaging activation study by PET in healthy participants suggested a role of the posterior parietal cortex, specifically of area PEG in the intraparietal sulcus, at the transition between the superior and the inferior parietal lobule, contralateral to the reaching upper limb (Clower et al., 1996). A following fMRI study showed the key involvement of the cerebellum, with different cerebellar regions being active in the early and late stages of PA (Küper et al., 2014). The assessment by event-related fMRI of the time course of neural activations during PA shows early neural activity in the anterior intraparietal sulcus, and in the parieto-occipital sulcus, respectively for error detection and error correction, as well as in the cerebellum, whose activity progressively increases; the superior temporal cortex becomes active in the later phase of PA (Luauté et al., 2009). Similar evidence was obtained in a following fMRI study, which confirms that neural activity in the cerebellum and in the posterior parietal cortex takes place in an early phase of PA (error correction), and increases in cerebellar regions, and in the inferior parietal lobule in a later phase, when spatial realignment occurs, along with the emergence of AEs; indeed, AEs are associated with increased activity in the right posterior cerebellum, and in the inferior parietal lobule, when right-handed participants adapted using their right hand (Chapman et al., 2010).

Studies in patients with left USN (rehabilitated with PA procedures using prisms displacing vision rightward, with leftward AEs) reveal preserved PA and AEs (Rossetti et al., 1998; Frassinetti et al., 2002a; Fortis et al., 2010). In five right brain-damaged patients with left USN, activations during a line bisection judgment task before and after one session of PA were assessed with PET (Luauté et al., 2006). The neural underpinnings of the prism-induced improvement of left USN at the task are featured by a bilateral activity involving: in the right hemisphere, the cerebellum and the posterior parietal cortex; in the left hemisphere, the thalamus and the temporo-occipital and medial temporal cortices (Luauté et al., 2006). Using fMRI, increased activation in intact bilateral parietal, frontal, and occipital cortices during bisection and visual search tasks has been found after PA in 7 patients with left USN (Saj et al., 2013). A recent study, in 12 right-brain-damaged patients (6 with left USN, and 6 without USN) and in 6 healthy participants, suggests that decreased activity in the right parietal association cortex during PA, and PA-induced reorganization of the right frontal and parietal areas may be responsible of USN improvements (Taniguchi et al., 2012). However, this last study used fNIRS (Functional Near-Infrared Spectroscopy), an imaging technique with lower spatial resolution and limited depth of recording, as compared to fMRI (Scarapicchia et al., 2017), and conclusions must be treated with much caution.

The involvement of a bilateral network is also suggested by evidence from a patient with bilateral damage to the superior parietal lobule and optic ataxia, who did not show any beneficial effects of PA on his defective lateral orienting (disengagement) of attention (Striemer et al., 2008). In another patient with bilateral posterior parietal damage and optic ataxia, preserved PA and AEs have been reported (Pisella et al., 2004). Left anterior cerebellar damage disrupts rightward AEs, when the patient wore prisms deviating the visual scene leftward, on the same side of the lesion, with both the left or the right hand having been used for PA by repeated pointings; leftward AEs after rightward deviations of the visual scene were preserved with both hands (Pisella et al., 2005). Another patient with a left cerebellar damage, and bilateral occipital damage, showed no leftward proprioceptive AEs after adaptation to prisms displacing rightward the visual scene; visual and visual-proprioceptive AEs were preserved. The leftward proprioceptive AEs, absent after PA alone, were temporarily restored by associating PA to a session of anodal transcranial direct current stimulation (tDCS) of the left posterior parietal cortex; anodal tDCS to the left cerebellum was partially effective (Calzolari et al., 2015). Finally, anodal tDCS of the damaged right posterior parietal cortex was shown to enhance the effects of PA rehabilitation of USN, while cathodal tDCS delivered to the left, intact, posterior parietal cortex wiped out the improvement of USN induced by PA (Làdavas et al., 2015).

In sum, evidence from imaging, lesion, and tDCS studies in both unimpaired and brain-damaged participants suggest a role of a vast bilateral cortico-cerebellar network activated by PA. Within this network, data from healthy participants appear to indicate a comparatively major role of the cerebellum in the later stage of spatial realignment (i.e., AEs), while the cortex, particularly the posterior parietal cortex, may contribute to the adaptation process both in its early and later (error detection and correction) stages. The extension of the bilateral cortical network supporting PA may account for the preservation of PA and AEs in patients with unilateral lesions, such as right-brain-damaged patients with USN (Rossetti et al., 1998; Frassinetti et al., 2002a; Fortis et al., 2010), as well as in one patient with bilateral cortical damage (Pisella et al., 2004).

Of interest, the PA network includes occipital, posterior parietal and temporal regions know to be involved in audio-visual interactions (Calvert et al., 2004; Huang and Sereno, 2018). It follows that the multisensory properties of the PA network might support crossmodal mechanisms of PA and AEs, as for instance adaptation to auditory targets, besides the time honored PA to visual targets. The present study aimed at verifying this hypothesis through three experiments exploring the influence of auditory and multisensory (audio-visual) stimuli on PA and its AEs, also considering the effect of a short-lasting PA phase, requiring fewer pointing movements, as compared the standard version.

## Experiment 1

### Aim

Experiment 1 explored whether PA performed through visuo-motor pointing movements toward auditory and audio-visual targets is effective as the standard visual paradigm. To this aim, participants, while looking through prismatic goggles deviating the visual field to the right, were required to perform a classic PA visuo-motor procedure in three different conditions requiring manual pointings to visual, auditory, or audio-visual targets; the three conditions were performed in separate sessions (one for each target modality). Proprioceptive, auditory-proprioceptive, visual-proprioceptive, and visual AEs were measured for each PA condition.

### Materials and Methods

#### Participants

Healthy volunteers (*N* = 24) participated in the experiment. Participants were students of the University of Milano-Bicocca (mean 23.3 years; *SD* = 1.4; range 21–28; *n* = 24; 12 female). All participants were right-handed, as assessed by the Edinburgh Inventory (Oldfield, 1971). All participants had normal or corrected-to-normal vision, reported no abnormalities in auditory perception, and had no history of neurological or psychiatric disorders. All participants were naïve as to the purpose of the experiment, were given course credits for their participation, and gave written informed consent. The study was conducted in accordance with the principles of the Declaration of Helsinki, and was approved by the Ethics Committee of the University of Milano-Bicocca.

#### Procedure

Participants underwent three sessions, in three different days, with an intersession interval of at least 24 h. Each session lasted about 1 h and included: (1) a pre-exposure phase, (2) a PA exposure phase, (3) a post-exposure phase, identical to the pre-exposure phase.

##### Pre- and post-exposure phases

In order to assess the presence and the magnitude of AEs, in each session participants performed 4 straight-ahead tests to measure the perception of the straight-ahead position (sagittal to their body midline) in the following modalities: proprioceptive, auditory-proprioceptive, visual-proprioceptive, and visual. In each session, each participant performed the 4 straight-ahead tests in the same order in the pre- and in the post-exposure phases. The tests’ order was counterbalanced across participants. For each test, 10 trials were given.

*Proprioceptive.* Each participant, seated in front of a table, with eyes closed, received instructions to point with the right index finger to the location on the table surface, perceived as the subjective straight-ahead. A graduated panel, aligned with the body’s midline, allowed the recording (degrees of visual angle in 1° steps) of the participant’s deviation from the objective body’s midline, with an accuracy of 0.5°.

*Auditory-proprioceptive.* In darkness, each participant received instructions to point with the right index finger to the location on the table surface subjectively perceived as the projection of a sound source; this procedure was based on an auditory open loop pointing task (Pavani et al., 2003; Michel et al., 2007). The sound consisted in a 1200 Hz tone, lasting 250 ms, emitted by a speaker placed 65 cm distant, and aligned with participant’s body-midline. No information was given to the participant about the location of the loudspeaker. A graduated panel, aligned with the body-midline, allowed the recording (degrees of visual angle in 1° steps) of the participant’s deviation from the straight-ahead position of the speaker, with an accuracy of 0.5°.

*Visual-proprioceptive.* In darkness, each participant received instructions to fixate a red LED placed in the straight-ahead position, 65 cm distant, and to point with the right index finger to the location on the table surface subjectively perceived as the projection of the light on the table. No information was given about the actual LED location, and a wooden box precluded participants from viewing the pointing movement, which then took place without any visual feedback. A graduated panel, aligned with the body-midline, allowed the recording (degrees of visual angle in 1° steps) of the participant’s deviation from the straight-ahead position of the LED, with an accuracy of 0.5°.

*Visual.* In darkness, each participant received instructions to stop verbally a red LED, moving horizontally just above eye level, at a distance of 65 cm from the participant’s mid-sagittal plane, when the light was perceived as straight-ahead. The 10 trials (5 with the light moving from the right to the left visual periphery, 5 from left to right) were given in a random fixed order. A ruler was fixed on the track edge of the apparatus facing the experimenter, to register the deviation of the visual judgment (cm, converted in degrees of visual angle) from the objective midline.

For each test, the mean deviation from the objective midline was calculated, both in the pre- and in the post- adaptation phase; positive values indicated a rightward deviation from the perceived body midline, negative values a leftward deviation.

##### PA exposure phase

In each session, participants adapted to an 11.4° rightward visual shift, induced by 20-dioptre, base-left prism glasses (Optique Peter, Lyon, France). Visuo-motor adaptation, which includes both a visual and an active proprioceptive-motor component, was achieved by the execution of 92 manual pointing movements toward a target presented at 4 different positions (+10°, +20° rightward, and -10° and -20° leftward, with respect to the participant’s body midline), in a pseudorandom fixed order, with the order being the same in the three sessions, for all participants. The target modality varied according to the adaptation condition of the session: visual (red LED), auditory (white noise burst), or audio-visual (simultaneous presentation of the LED and of the white noise burst). In each session, only one target modality was presented, and the order of the three adaptation conditions was counterbalanced across participants. Each stimulus was presented for 150 ms (Frassinetti et al., 2002c).

The apparatus for the presentation of the target stimuli was adapted from Frassinetti et al. (2002c). Four red LEDs and four piezoelectric loudspeakers were mounted in couple, on a semicircular black board, arranged horizontally at the participant’s ear level, and located at an eccentricity of 10° and 20° to the left and the right of the center of the apparatus, which was aligned with the participant’s body midline. Participants were unable to see the loudspeakers mounted behind the board. Participants had received instructions to point with their right fingertip to the target, with a fast and accurate movement, and then to return to the initial position (right finger on the sternum). The view of the pointing movement was occluded by means of a wooden box and a cape that covered the participant’s arms, with the finger becoming visible at the very last part of the movement (Redding et al., 2005). The external side of the wooden box, facing the experimenter, was graduated in degrees of visual angle to allow the recording of the deviation of each pointing movement from the target with an accuracy of 1°. Rightward deviations from the target were scored with positive values, leftward deviations with negative values. The pointing adaptation procedure lasted about 20 min.

#### Statistical Analyses

Statistical analyses were carried out with SPSS (IBM SPSS Statistic version 21). In all ANOVAs, significant effects and interactions were explored with Bonferroni *post hoc* test for multiple comparisons. Significance was set at *α* = 0.05. To quantify the magnitude of the reported effects, partial eta squared ($\eta_{p}^{2}$) values for *F*-tests (Cohen, 1988) are provided.

The presence of PA (i.e., the reduction of the initial pointing error) was assessed via a 2-factor repeated-measures ANOVA (Winer, 1971), performed on the mean pointing deviations from the target in the first (1–4 averaged), middle (45–48), and last (89–92) four trials, with the within-subjects main factors *target modality* (visual, auditory, audio-visual), and *trial* (first, middle, last).

In order to assess the presence of sensorimotor AEs in the proprioceptive, auditory-proprioceptive, and visual-proprioceptive straight-ahead tests, a repeated-measures ANOVA was run on the participants’ mean straight-ahead performance, with the within-subjects main factors *target modality* (visual, auditory, audio-visual), *time* (pre-PA post-PA), and *test* (proprioceptive, auditory-proprioceptive, visual-proprioceptive). A separate repeated-measures ANOVA was performed on the visual test, with the within-subjects main factors *target modality* (visual, auditory, audio-visual), and *time* (pre-PA post-PA). The visual test was kept separate from the other tests, as a shift in the opposite direction was expected, based on previous studies (Redding and Wallace, 2010).

### Results

#### Prism Adaptation

**FIGURE 1:**
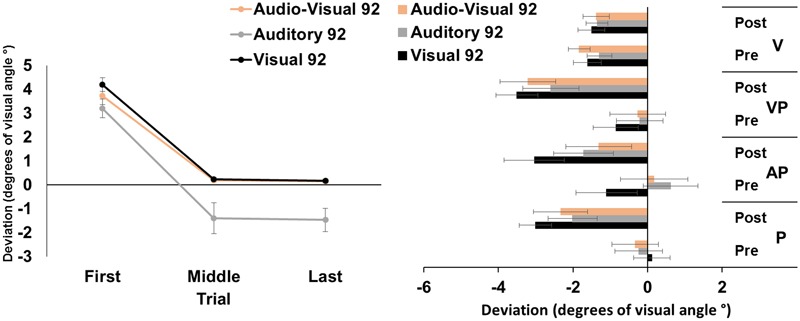
Results from Experiment 1. **(Left)** Mean (Standard Error, SE) deviation of the pointing movements (average of first, middle, and last four pointings) of the right index finger from the target in the 92-pointings adaptation task in the visual (black), auditory (gray), and audio-visual (orange) conditions, in degrees of visual angle (°); positive values correspond to rightward deviations from the targets, negative values to leftward deviations. **(Right)** Mean (SE) deviation from the body midline in the proprioceptive (P), auditory-proprioceptive (AP), visual-proprioceptive (VP), and visual (V) straight-ahead tests, before (pre), and after (post), the visual (black), auditory (gray), and audio-visual (orange) conditions of adaptation, in degrees of visual angle (°); positive values correspond to rightward deviations from the perceived body midline, negative values to leftward deviations.

The ANOVA run on the average pointing errors showed significant main effects of target modality [*F*_(2,46)_ = 8.36, *p* < 0.001, $\eta_{p}^{2}$ = 0.27], and of trial [*F*_(2,46)_ = 153.39, *p* < 0.001, $\eta_{p}^{2}$ = 0.87]; the interaction target modality by trial was not significant [*F*_(4,92)_ = 1.78, *p* = 0.14]. *Post hoc* comparisons for the main effect of target modality showed that the pointing movements toward visual and audio-visual targets were more deviated to the right, as compared to those to auditory targets (both *p-values* < 0.05). The main effect of trial showed that, across modalities, pointings in the first trials were more rightward deviated, as compared to pointings both in the middle, and in the last trials (both *p-values* < 0.001). Average middle and last pointings did not differ from each other (*p* = 1), demonstrating the occurrence of adaptation in all modalities after only 45 trials (**Figure 1**, left panel).

#### Aftereffects

The ANOVA showed a significant main effect of time [*F*_(1,23)_ = 47.80, *p* < 0.001, $\eta_{p}^{2}$ = 0.67]. Specifically, the mean straight-ahead post-adaptation deviation (-2.53 ± 2.90°, M ± SD°) was more shifted to the left as compared to the pre-adaptation deviation (-0.23 ± 2.69°) (**Figure 1**, right panel). No other main effect or interaction was significant [modality: *F*_(2,46)_ = 2.44, *p* = 0.10; test: *F*_(2,46)_ = 2.59, *p* = 0.09; modality by time: *F*_(2,46)_ = 0.41, *p* = 0.66; modality by test: *F*_(4,92)_ = 2.33, *p* = 0.06; time by test: *F*_(2,46)_ = 1.61, *p* = 0.21; modality by time by test: *F*_(4,92)_ = 1.84, *p* = 0.13]. The ANOVA on the visual test did not show any significant main effect or interaction [modality: *F*_(2,46)_ = 0.48, *p* = 0.62; time: *F*_(1,23)_ = 0.72, *p* = 0.41; modality by time: *F*_(2,46)_ = 0.80, *p* = 0.45].

### Discussion

These findings demonstrate that PA, obtained by repeated visuo-motor pointings to targets in the visual, auditory, or audio-visual modalities, brings about comparable amounts of AEs in the proprioceptive, auditory-proprioceptive, and visual-proprioceptive straight-ahead tests. No effect is found in the visual straight-ahead test. Moreover, results on PA, as indexed by the correction of the pointing error, show that, firstly, participants reduce their pointing error (i.e., the last pointings are significantly less rightward deviated compared to the first pointings) in all the three PA conditions. This result, expected for the classic visuo-motor PA to the visual target, is a novel finding for the other two modalities. Secondly, pointings to auditory targets are overall more leftward deviated, as compared to pointings to visual, and audio-visual targets. And, thirdly, participants reduce significantly their pointing error after only 45 trials, in all modality conditions.

## Experiment 2

### Aim

Following the findings of Experiment 1, in Experiment 2 we aimed at assessing whether reducing considerably the number of trials in the adaptation phase would bring about both PA (i.e., error correction), and AEs. Thus, in Experiment 2 the number of trials was reduced from 92 to 24, to assess whether PA and AEs took place also with fewer trials. We investigated the effect of a reduced number of trials (i.e., 24 vs. 92) both in the classic visual target condition, and in the audio-visual condition which, in Experiment 1, brought about a comparable pattern of adaptation (in terms of error reduction in middle and last pointing trials). The choice to exclude the unimodal auditory modality in Experiment 2 was further driven by the aim of exploring whether the different pattern of results in the adaptation phase for the auditory modality, found in Experiment 1, could have been due to the presence of the four LEDs on the apparatus. In fact, although these were never activated during the unimodal auditory condition, they might nevertheless have provided visual cues to the participants’ pointing movement. The unimodal auditory modality was instead investigated in the following Experiment 3, modifying the adaptation apparatus in such a way that no external visual cues could have biased the participant’s pointing movement.

### Materials and Methods

#### Participants

Healthy volunteers (*N* = 24) participated in the experiment (mean 25.0 years; *SD* = 3.1; range 20–31; *n* = 24; 12 female).

#### Procedure

Methods of the pre- and post-adaptation phases were similar to those of Experiment 1. In the adaptation phase, instead, participants underwent only two sessions (instead of three), in two separate days, one with an adaptation phase to 24 visual targets, and the other with an adaptation phase to 24 audio-visual targets.

#### Statistical Analyses

Prism adaptation was assessed via a 2-factor repeated-measures ANOVA, performed on the mean deviations from the target in the first (1–4), middle (11–14), and last four (21–24) trials, with the within-subjects factors *target modality* (visual, audio-visual), and *trial* (first, middle, last). Sensorimotor AEs were assessed as in Experiment 1, with the difference that target modality had two levels (visual, audio-visual).

### Results

#### Prism Adaptation

**FIGURE 2:**
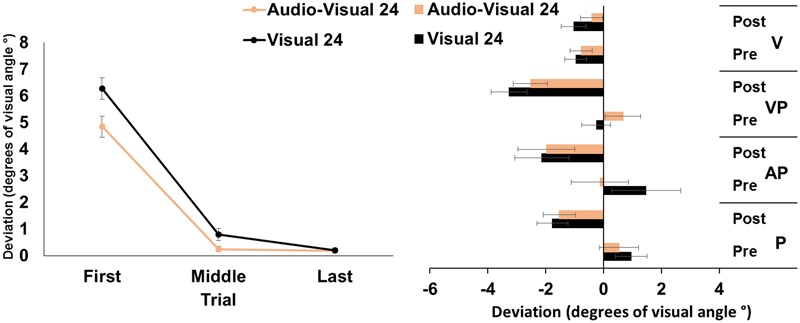
Results from Experiment 2. **(Left)** Mean (SE) deviation of the pointing movements (average of first, middle, and last four pointings) of the right index finger from the target in the 24-pointings adaptation task in the visual (black), and audio-visual (orange) conditions, in degrees of visual angle (°); positive values correspond to rightward deviations from the targets, negative values to leftward deviations. **(Right)** Mean (SE) deviation from the body midline in the proprioceptive (P), auditory-proprioceptive (AP), visual-proprioceptive (VP), and visual (V) straight-ahead tests, before (pre), and after (post), the visual (black), and audio-visual (orange) conditions of adaptation, in degrees of visual angle (°); positive values correspond to rightward deviations from the perceived body midline, negative values to leftward deviations.

The ANOVA run on the mean deviation during the PA exposure showed significant main effects of target modality [*F*_(1,23)_ = 9.12, *p* < 0.01, $\eta_{p}^{2}$ = 0.28], and of trial [*F*_(2,46)_ = 272.80, *p* < 0.001, $\eta_{p}^{2}$ = 0.92]; the target modality by trial interaction was significant [*F*_(2,46)_ = 4.79, *p* < 0.05, $\eta_{p}^{2}$ = 0.12]. *Post hoc* comparisons for the interaction showed that, in both the visual and in the audio-visual conditions, the first pointing movements were more rightward deviated (all *p-values* < 0.001) than the middle, and the last pointing movements, which were more accurately directed at the target. Moreover, in the visual condition, the middle pointings were more rightward deviated, compared to the last pointings (*p* < 0.05), while in the audio-visual condition the middle pointings did not differ from the last ones (*p* = 1). Finally, the first and the middle pointings in the visual condition were more rightward deviated, compared to pointings to audio-visual targets (both *p-values* < 0.05), while the last pointings in the two conditions did not differ (*p* = 95) (**Figure 2**, left panel).

#### Aftereffects

The ANOVA showed a significant main effect of time [*F*_(1,23)_ = 37.51, *p* < 0.001, $\eta_{p}^{2}$ = 0.62]. Specifically, overall, the mean straight-ahead post-adaptation deviation (-2.20 ± 2.73°) was significantly more shifted to the left, as compared to the pre-adaptation deviation (0.54 ± 2.21°) (**Figure 2**, right panel). No other main effect or interaction reached the significance level [modality: *F*_(1,23)_ = 0.00, *p* = 0.99; test: *F*_(2,46)_ = 1.46, *p* = 0.24; modality by time: *F*_(1,23)_ = 0.88, *p* = 0.36; modality by test: *F*_(4,92)_ = 1.73, *p* = 0.19; time by test: *F*_(2,46)_ = 0.56, *p* = 0.58; modality by time by test: *F*_(2,46)_ = 0.96, *p* = 0.39], thus demonstrating that the two adaptation conditions brought about the same amount of AEs in the proprioceptive, auditory-proprioceptive, and visual-proprioceptive straight-ahead tests. The ANOVA on the visual test did not show any significant main effect or interaction [modality: *F*_(1,23)_ = 1.45, *p* = 0.24; time: *F*_(1,23)_ = 0.60, *p* = 0.44; modality by time: *F*_(1,23)_ = 2.89, *p* = 0.10].

### Discussion

These findings demonstrate that PA obtained by a reduced number of visuo-motor pointings to targets in the visual or audio-visual modality, brought about comparable amounts of AEs in the proprioceptive, auditory-proprioceptive, and visual-proprioceptive straight-ahead tests. No effect was found in the visual straight-ahead test. Results about PA show, firstly, a reduction of the pointing error (i.e., the last pointings are significantly less rightward deviated than the first pointings) in both the visual and in the audio-visual target adaptation conditions. Secondly, participants show a smaller pointing error in the first and middle trials in the audio-visual target condition than in the visual condition; the pointing error further diminishes in the visual target condition from the middle to the last trials, in which it becomes not different from that of the audio-visual target condition.

## Experiment 3

### Aim

The influence of the number of trials with unimodal auditory targets necessary for inducing reliable PA and AEs was assessed.

### Participants

Healthy volunteers (*N* = 24) participated in the experiment (mean 24.5 years; *SD* = 2.0; range 20–31; *n* = 24; 12 female).

### Procedure

Methods of the pre- and post-adaptation phases were similar to those of Experiment 1 and Experiment 2. In the adaptation phase, instead, participants underwent two sessions, in two separate days: one session with an adaptation phase to 92 auditory targets, and the other session with an adaptation phase to 24 auditory targets. Moreover, in this experiment, the LEDs were covered and hidden to the participants’ sight, so that the pointing movement to auditory targets could only rely on sound localization, with no visual cues.

### Statistical Analyses

Prism adaptation was assessed via a 2-factor repeated-measures ANOVA, performed on the mean deviations from the target in the first (1–4), middle (45–48 for the 92-targets condition, 11–14 for the 24-targets condition), and last (89–92 for the 92-targets condition, and 21–24 for the 24 targets condition) four pointing movements, with the within-subjects factors *number of targets* (92, 24) and *trial* (first, middle, last). Sensorimotor AEs were assessed as in Experiment 1, with the within-subjects factor *number of targets* (92, 24), instead of target modality.

### Results

#### Prism Adaptation

**FIGURE 3:**
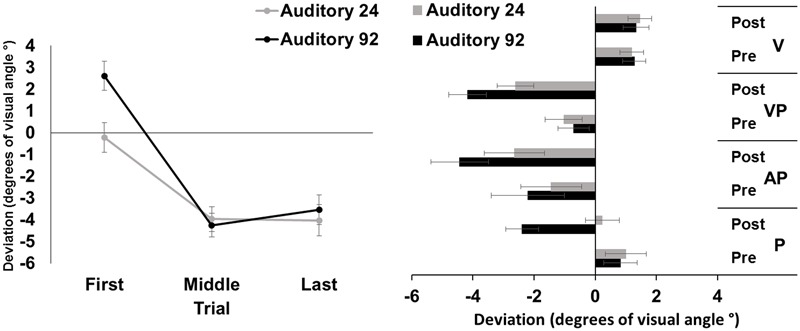
Results from Experiment 3. **(Left)** Mean (SE) deviation of the pointing movements (average of first, middle, and last four pointings) of the right index finger from the target in the 92-pointings (black) and in the 24-pointings (gray) adaptation conditions to auditory targets, in degrees of visual angle (°); positive values correspond to rightward deviations from the auditory targets, negative values to leftward deviations. **(Right)** Mean (SE) deviation from the body midline in the proprioceptive (P), auditory-proprioceptive (AP), visual-proprioceptive (VP), and visual (V) straight-ahead tests, before (pre), and after (post), the 92-pointings (black), and the 24-pointings (gray) adaptation conditions to auditory targets, in degrees of visual angle (°); positive values correspond to rightward deviations from the perceived body midline, negative values to leftward deviations.

The repeated measures ANOVA showed a significant main effect of trial [*F*_(2,46)_ = 51.37, *p* < 0.001, $\eta_{p}^{2}$ = 0.69], while the main effect of number of targets was not significant [*F*_(1,23)_ = 3.93, *p* = 0.06]. The interaction number of targets by trial was significant [*F*_(2,46)_ = 48.29, *p* < 0.001, $\eta_{p}^{2}$ = 0.26]. *Post hoc* comparisons for the interaction showed that in both the 92- and the 24-targets conditions, the average deviations of the first pointings were more rightward, as compared to both the middle, and the last pointings (all *p-values* < 0.001); the average middle and last pointing deviations were both directed to the left of the target to the same extent (both *p-values* > .65). The average pointing error in the first trials of the 24-trial condition was more leftward deviated, as compared to that of the 92-trial condition (*p* < 0.001) (**Figure 3**, left panel).

#### Aftereffects

The ANOVA showed significant main effects of number of targets [*F*_(1,23)_ = 7.23, *p* < 0.05, $\eta_{p}^{2}$ = 0.24], of time [*F*_(1,23)_ = 32.56, *p* < 0.001, $\eta_{p}^{2}$ = 0.59], and of test [*F*_(2,46)_ = 6.05, *p* < 0.01, $\eta_{p}^{2}$ = 0.21]. The interaction number of targets by time was significant [F_(1,23)_ = 7.88, *p* = 0.01, $\eta_{p}^{2}$ = 0.25]. *Post hoc* comparisons for the main effect of test showed that, overall, performance in the proprioceptive test (-0.08 ± 2.39°) was less leftward deviated as compared to that of both the auditory-proprioceptive (*p* < 0.05), and the visual-proprioceptive (*p* < 0.01) straight-ahead tests. *Post hoc* comparisons for the interaction showed that both in the 92-targets (*p* < 0.001), and in the 24-targets (*p* < 0.05) conditions, the average post-adaptation deviations were more leftward deviated as compared to pre-adaptation deviations in the straight-ahead tests. Moreover, while the pre-adaptation deviations did not differ in the two conditions (*p* = 0.72), after the 92-targets condition, the post-adaptation deviations were more leftward deviated (*p* < 0.001) as compared to those after the 24-targets condition (**Figure 3**, right panel). These comparisons thus demonstrate that both adaptations brought about AEs in the proprioceptive, auditory-proprioceptive, and visual-proprioceptive straight-ahead tests, but that these AEs were greater after adapting with 92 trials than with 24 trials.

The ANOVA on the visual test did not show any significant main effect or interaction [number of targets: *F*_(1,23)_ = 0.00, *p* = 0.95; time: *F*_(1,23)_ = 0.57, *p* = 0.46; number of targets by time: *F*_(1,23)_ = 0.42, *p* = 0.52].

### Discussion

These findings demonstrate that PA obtained with visuo-motor pointings to unimodal auditory targets, eliminating any visual cues about their location in the visual field, cause reliable AEs in the proprioceptive, auditory-proprioceptive, and visual-proprioceptive straight-ahead tasks. However, AEs of a smaller amplitude occur after PA with a reduced number of pointings (24) to auditory targets, as compared to those taking place with 92 pointings. No effect was found in the visual straight-ahead test. Results on PA, as indexed by the correction of the pointing error, show that participants improve their pointing accuracy, with their last pointings being significantly less rightward deviated than their first pointings in both adaptation conditions; however, accuracy in the first pointings of the 24 trials condition was already greater (namely it showed a minor rightward deviation), as compared to that observed in the 92 pointing condition.

## General Discussion

In three experiments, the influence of targets presented in different sensory modalities was investigated. Visual, which is the classic target modality for PA (Redding et al., 2005; Redding and Wallace, 2010), auditory, and audio-visual targets were used. The number of trials of the exposure phase was varied, comparing the 92-pointings PA procedure, broadly matching the typical number of trials used in previous studies (e.g., 90 pointings: Frassinetti et al., 2002a; Fortis et al., 2010; see also Rossetti et al., 1998, 50 pointings; Striemer et al., 2016, 200 pointings) with a shortened 24-pointings procedure.

Results from Experiment 1 show that participants are able to reduce their pointing error during the 92-trials adaptation phase, regardless of the target modality toward which the pointing movements are directed. Overall, pointings to auditory targets are more deviated to the left of the target, as compared to those to visual and audio-visual ones, which, instead, are more rightward deviated. These effects are accompanied by reliable AEs in the proprioceptive, auditory-proprioceptive, and visual-proprioceptive straight-ahead tests. Results of Experiment 2 show that even a shortened PA phase requiring 24 pointings is sufficient to bring about AEs in the proprioceptive, auditory-proprioceptive, and visual-proprioceptive tests, after adaptation to prismatic displacement with both visual and audio-visual targets. Secondly, in both the first and the middle trials of the adaptation phase, pointings toward audio-visual targets are more accurate than those to purely visual targets, suggesting that participants are more precise and quicker in correcting the pointing error, with bimodal, audio-visual, targets, as compared to unimodal, visual, ones. These novel findings suggest that multisensory audio-visual targets, as compared with unimodal (visual) ones, may fasten adaptation in the first trials. Results from Experiment 3 further confirm that pointings to purely unimodal auditory targets, with no visual clues about their spatial location, are still effective in bringing about AEs in the proprioceptive, auditory-proprioceptive, and visual-proprioceptive straight-ahead tests. However, PA with a reduced number of pointings (24) to auditory targets brings about AEs of a smaller amplitude, as compared to those taking place with 92-pointings.

### Adaptation and Aftereffects

#### Adaptation as a Reduction of the Pointing Error

One first novel finding of this study is that participants are able to adapt and correct the initial pointing error during prism exposure, by pointing to auditory unimodal targets (Experiments 1 and 3). In this condition, participants point to the sound source, which is not visible, being hidden behind the apparatus. The only visual feedback provided is the sight of the participants’ right index finger, emerging at the opposite side of the apparatus, at the end of the movement. However, no visual cue about the position of their finger relative to the target is available. This may prevent, or at least reduce, the participants’ awareness of the error resulting from the immediate ‘direct effect’ of prismatic displacement, thus minimizing the role of the recalibration process. Recalibration is considered a top–down, strategic and voluntary component of PA, subtending the main process responsible of the early correction of the pointing error (Redding et al., 2005). If this is the case, the suggestion can be made that PA to auditory targets relies mainly on the more automatic processes of adaptation, namely the spatial visuo-motor realignment. This process contributes both to the error reduction in the later stages of exposure, and to the development of AEs. This proposal is supported by evidence showing that AEs may be obtained by means of PA paradigms that substantially reduce, or totally eliminate, the participants’ awareness of any discrete error to correct during the movement. This may be achieved by adopting procedures in which participants are involved in visuo-motor activities, more ecological than the repeated pointings method, typically used in PA paradigms. These activities (Shiraishi et al., 2008; Fortis et al., 2010) have been successfully used in the rehabilitation of USN. Capitalizing on the observation that right brain-damaged patients with left USN “*exhibit both a lack of awareness for the spatial distortions imposed during visuomanual PA procedures, and exaggerated post-adaptation… AEs*,” Michel et al. (2007) report that the gradual exposure to the prism-induced visual displacement prevents participants from becoming aware of the optical deviation. A peculiarity of our findings regarding PA to auditory targets is that, in this condition, the middle and last pointings are characterized by an error to the left of the target, rather than being correctly directed to it, as it occurs instead in the visual and audio-visual conditions. This effect may be due, on one hand, to a greater difficulty of auditory spatial localization, as compared to visual and audio-visual localization (Stein et al., 1989; King, 2009; Martin et al., 2015), and, on the other hand, to the persistence of recalibration strategies in the face of the developing of spatial realignment, which may result in overcompensation, namely a pointing error to the left of the target (Redding et al., 2005).

A second main finding of this study is that participants adapt more quickly, being more precise in pointing to audio-visual targets, as compared to visual ones (Experiment 2). This finding suggests a possible multisensory-induced facilitating effect of spatial localization and manual pointing to the target, which follows the interaction of auditory and visual information (Stein et al., 1989; King, 2009; Martin et al., 2015). It may be objected to this interpretation that the same effect was not found in Experiment 1, where the middle pointings of the visual and audio-visual conditions were equally directed to the target. However, it is worth noting that the entire exposure phase of Experiment 2, lasting 24 trials, was shorter than the first half of the exposure phase of Experiment 1 (92 pointings in total). As found by Fortis et al. (2010), the reduction of the pointing error occurs within the first 45 trials in a standard 90-poitings paradigm. Thus, whenever any multisensory facilitation is present, this is likely to have occurred before the middle trials used in Experiment 1 (i.e., trials number 45–48). The analysis of the error reduction over time of the shortened PA version in Experiment 2 reveals that, in the visual condition, halfway through the exposure phase (i.e., trials 11–14), participants are yet not able to point correctly to the target, while this is the case in the audio-visual condition. Also, even in the visual condition, 24 targets are sufficient to achieve a complete pointing error reduction, given that the last pointings of both conditions are correctly directed to the targets. Importantly, this multisensory facilitating effect is confined to the pointing error reduction process, and does not extend to the building up of larger AEs. Studies in neurologically unimpaired participants indicate that adaptation and AEs are highly correlated (Fernaìndez-Ruiz and Diìaz, 1999). However, dissociations have been found in one brain-damaged patient (Calzolari et al., 2015), and occur in physiological aging (Fernández-Ruiz et al., 2000), suggesting that the two processes implied in PA (the error reduction and the production of sensory-motor AEs), are, at least partially, independent.

#### Aftereffects

Adaptation to auditory targets produces a pattern of AEs comparable to that occurring after the standard PA procedure to visual targets, in terms of both the involved sensory systems, and their amplitude (Experiment 1). Firstly, this evidence further supports the view that awareness of the pointing error is not essential for the building up of AEs (Michel et al., 2007; Shiraishi et al., 2008; Fortis et al., 2013). Secondly, the conclusion can be drawn that cross-modal effects take place in PA, with adaptation obtained through pointings to targets presented in the auditory modality transferring to the other sensory systems involved in the adaptation process (i.e., the proprioceptive, and the visual systems).

A number of investigations in healthy participants shows cross-modal effects in the opposite direction, namely: effects in the auditory domain by the displacement of the visual scene brought about by prisms. The auditory midline, when participants (with eyes open) wear prisms shifting the visual scene leftward is displaced in opposite directions before and after adaptation (Lackner, 1973). Healthy participants, after wearing up to 4 h optical prisms displacing the visual scene leftward or rightward, show shifts in sound localization in the same direction of the prism-induced deviation, confirming the cross-modal modulation on auditory space perception induced by PA to visual targets (Cui et al., 2008). Also, in healthy participants, adaptation to prisms displacing the visual scene leftward and rightward modulates auditory time processing, as assessed by a time bisection task, with an overestimation effect associated with rightward AEs (Magnani et al., 2012). Moreover, effects of a classic PA session to visual stimuli on the auditory modality have also been assessed in studies with USN patients. Left auditory extinction is definitely improved (Jacquin-Courtois et al., 2010), and marginally significant improvements have been found in an auditory dual task (Eramudugolla et al., 2010). Conversely, auditory tones and alerting sounds induce short-term improvements of visuo-spatial attention deficits in patients with USN (Robertson et al., 1998; Frassinetti et al., 2005; Van Vleet and Robertson, 2006).

Importantly, the cross-modal transfer from PA to unimodal auditory targets to tasks that do not include the auditory modality (visual and proprioceptive) occurs in a complete fashion in both the 92-pointings and the 24-pointings conditions. However, in the reduced 24-trials version, AEs are smaller (Experiment 3), suggesting that the auditory component of the PA process may be less central to the building up of the AEs, than the sensorimotor pointing activity *per se*.

Taken together, the present evidence suggests that visuo-motor PA with auditory targets may rely on cross-modal plasticity processes that are more automatic and do not require a complete awareness of the spatial mismatch between sensory modalities, as compared to those acting when participants point to visual or audio-visual targets.

In all experiments, AEs in the visual straight-ahead test were not found. These findings are in line with evidence showing an inconsistent occurrence of visual AEs, especially as compared to AEs in tasks that involve also a proprioceptive and motor response, such as the proprioceptive and visuo-proprioceptive straight-ahead tests, or, for the visuospatial domain, the line bisection task (Ronchi et al., 2011; Striemer et al., 2016). Indeed, the proprioceptive straight-ahead has been considered the most sensitive and reliable measure of AEs after PA (Rossetti et al., 1998; Ronchi et al., 2011).

#### Putative Neural Correlates

Differences, related to the modality of the target to which participants point, are found in the adaptation stage, but not in the subsequent AEs, which are comparable across target modalities. Crossmodal effects confined to the adaptation (pointing) stage suggest a putative main cortical, rather than cerebellar, involvement. One such cortical candidate is the posterior parietal cortex, involved both in the adaptation process, as reviewed in the introduction, and in cross-modal integration of different input modalities (Calvert et al., 2004; Huang and Sereno, 2018).

### Number of Trials and Clinical Implications

Other relevant findings from our study concern evidence about the number of pointing movements necessary to obtain AEs. The optimal parameters of PA, including exposure duration, number of targets during exposure, and number of pointing movements, have been considered in a number of studies performed in healthy participants (Colent et al., 2000; Girardi et al., 2004; Bultitude and Woods, 2010; Jacquin-Courtois et al., 2013). More than one target should be used, and the minimal number of exposure trials has been suggested to be around 60 (Jacquin-Courtois et al., 2013). In a rehabilitation study of USN in right-brain-damaged patients, using a standard 90-pointings paradigm (see Frassinetti et al., 2002a), Fortis et al. (2010) found, however, that the reduction of the pointing error occurs within the first 45 trials.

A parameter such as the number of trials has relevant implications for rehabilitation purposes. Increasing the patients’ compliance to the treatment, by reducing its duration, could increase their motivation, attention and involvement in the treatment session. The level of satisfaction, and the possible difficulties in performing the classic 90 repeated pointings vs. the ecological adaptation procedure (Fortis et al., 2010), have been evaluated and compared in healthy participants, with the ecological procedure being rated as more pleasant, less monotonous, and more sustainable than the pointing procedure (Fortis et al., 2013). Reducing the duration of the repeated pointing procedure, by diminishing the number of trials, may result in a more sustainable and less tedious task, increasing the patients’ compliance to the treatment. Our results show, in healthy individuals, that drastically reducing the number of trials (i.e., from 92 to 24 pointings) is sufficient for reducing the pointing error, as well as for inducing the expected AEs. This evidence may have important implications for the rehabilitation of USN patients: visuo-motor adaptation to auditory targets may be used as a supplementary or compensatory cross-sensory strategy for the treatment of the visual deficits, that these patients may also show (see e.g., Vallar and Perani, 1986; Stone et al., 1998).

## Author Contributions

EC, FA, NB, and GV designed research; EC and FA performed research; EC and FA analyzed the data; and EC, FA, NB, and GV wrote the paper.

## Conflict of Interest Statement

The authors declare that the research was conducted in the absence of any commercial or financial relationships that could be construed as a potential conflict of interest.
